# Supplementary material for: Identification of interferon-stimulated genes that attenuate Ebola virus infection
Source: Nat Commun. 2020 Jun 11;11:2953. doi: 10.1038/s41467-020-16768-7 (PMC7289892; doi:10.1038/s41467-020-16768-7)
Supplement: Supplementary file 1 — Supplementary Information [file 41467_2020_16768_MOESM1_ESM.pdf]

Supplementary Information

Identification of Interferon-Stimulated Genes that Attenuate Ebola Virus Infection

Kuroda *et al.*

Supplementary Figures

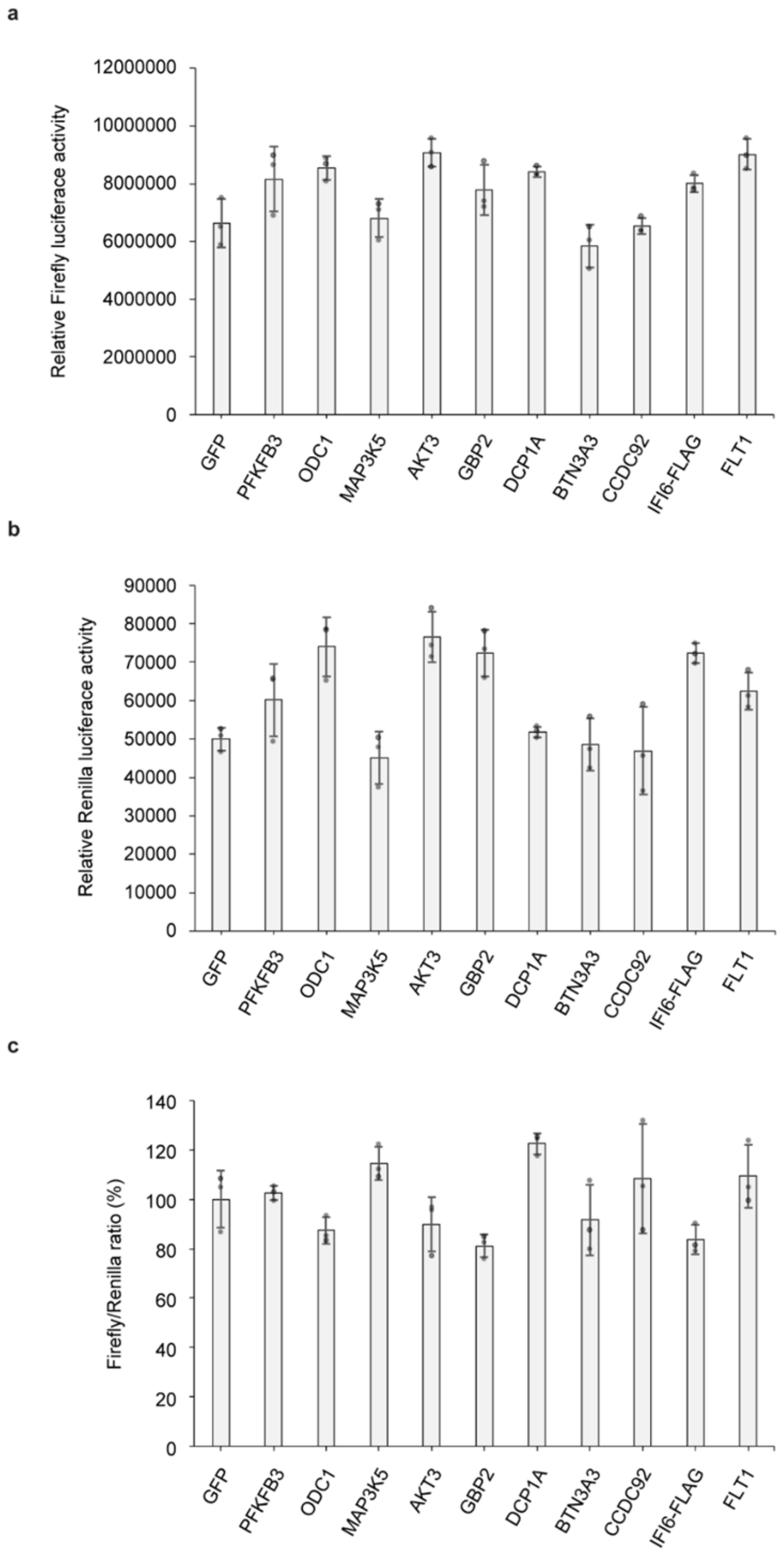

Supplementary Figure 1. Effect of ISGs on firefly and Renilla luciferase activities. HEK-293T cells were transfected with vectors expressing firefly luciferase and Renilla luciferase along with each of the indicated ISGs. At 24 h post-transfection, cell lysates were collected and subjected to the dual-luciferase reporter assay. Relative activities of (**a**) firefly luciferase, (**b**) Renilla luciferase, and (**c**) firefly/Renilla luciferase are presented

as means ± SD. Data are representative of four independent experiments. Source data are provided as a Source Data file.

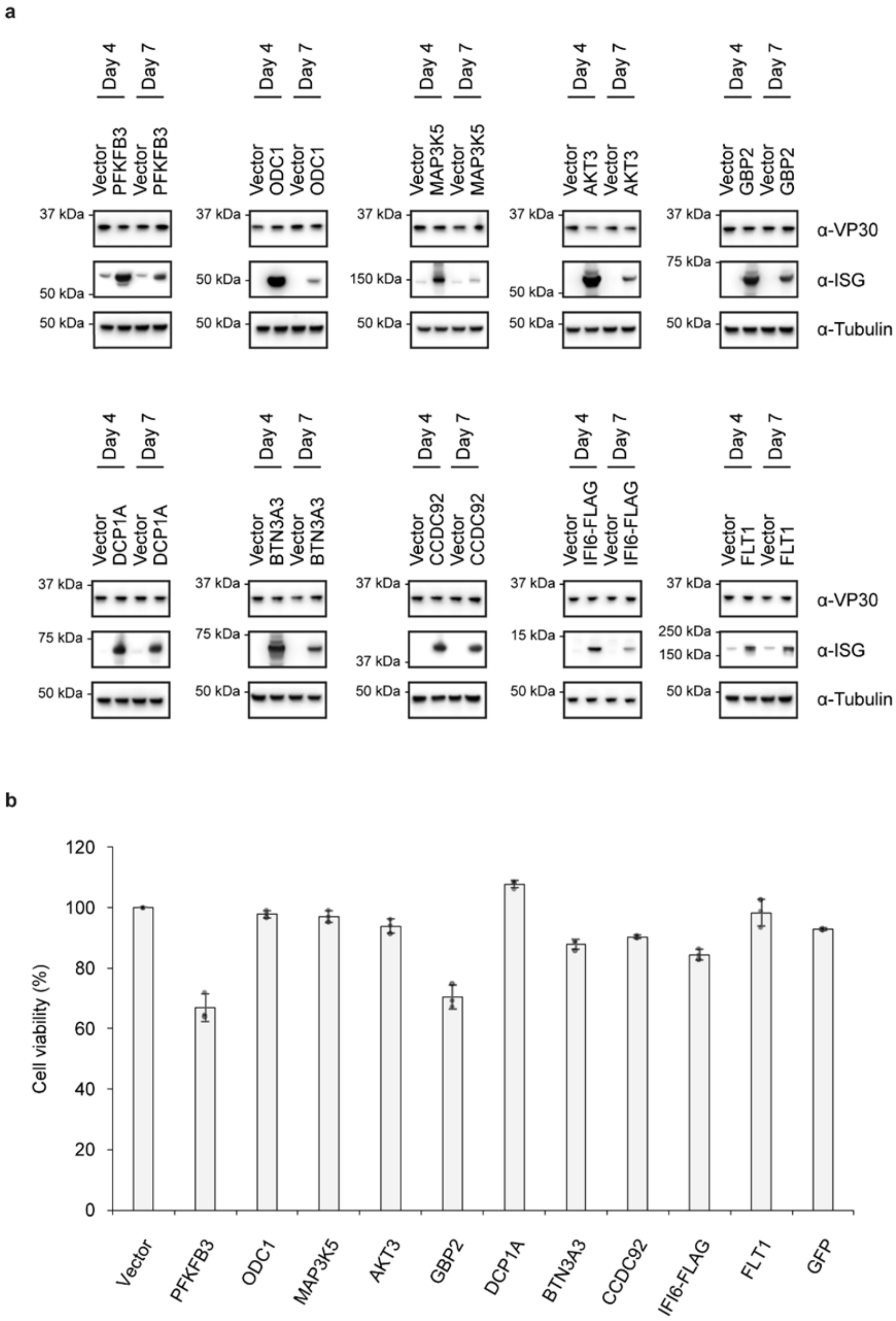

Supplementary Figure 2. ISG expression and its effect on cell viability. (**a**) HEK-293T VP30 cells were transfected with vectors expressing the indicated genes. On days 4 and 7 post-transfection, expression of each ISG and VP30 was confirmed by western blot analysis. (**b**) HEK-293T VP30 cells were transfected with vectors expressing the indicated genes and then infected with EBOVΔVP30-GFP. On day 6 post-infection, cell viability was measured by using a cell proliferation assay. Data are presented as means ± SD, and are representative of three independent experiments. Source data are provided as a Source Data file.

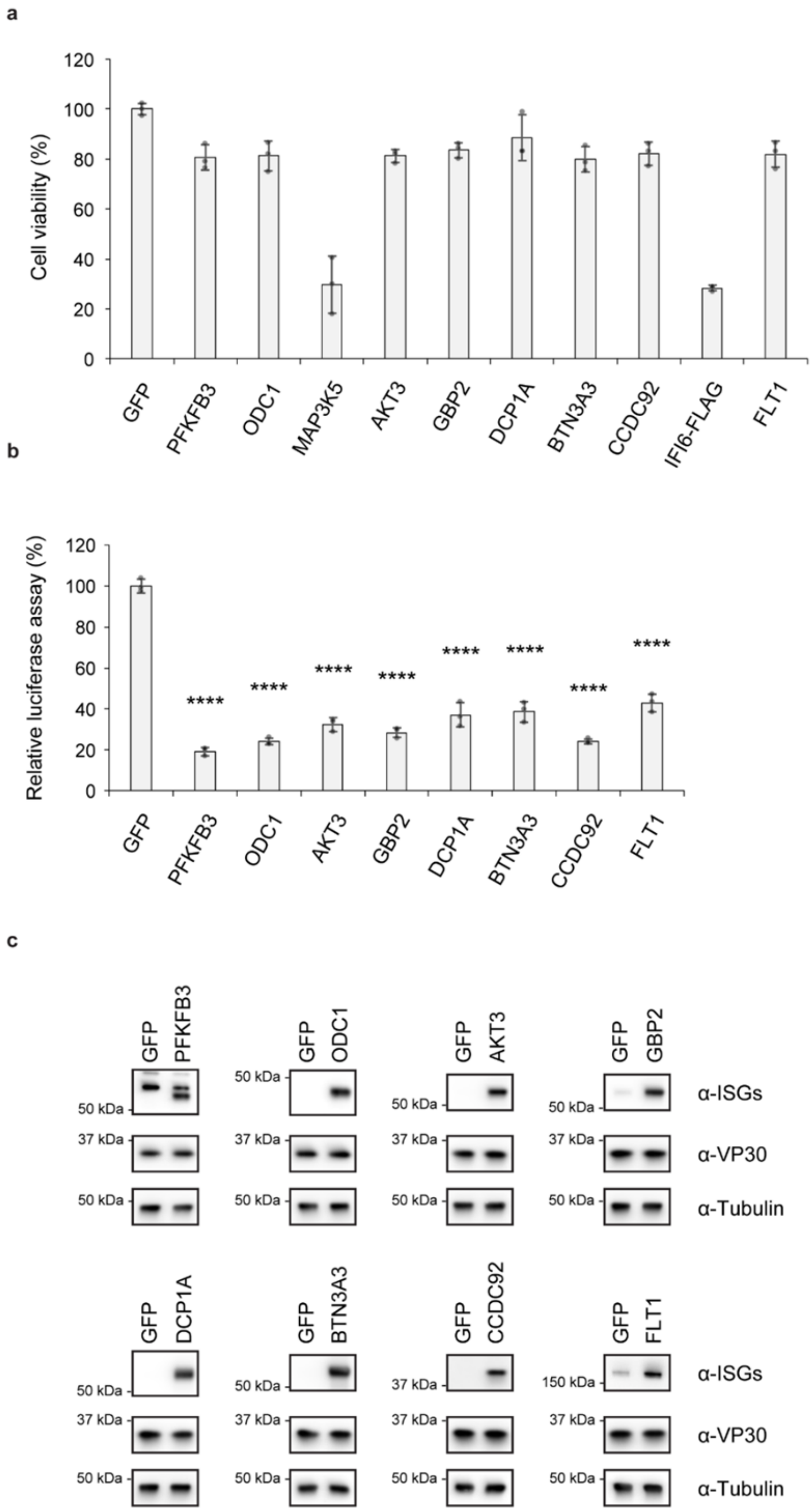

Supplementary Figure 3. Effect of ISGs on EBOV-driven luciferase activity in primary cells. HUVEC VP30 cells were transfected with vectors expressing the indicated genes. (**a**) At 24 h post-transfection, cell lysates were collected and subjected to a cell viability assay. (**b**) Transfected cells with >75% cell viability were infected with EBOVΔVP30-luc, and virus-driven Renilla luciferase activity was measured on day 3 post-infection. (**c**) Western blot analysis was performed with cell lysates to confirm the expression of each ISG and VP30. Data are presented as means ± SD, and are representative of three independent experiments. (*) indicates a statistically significant difference (*p* values of two-tailed Student's *t*-tests; ****$p < 0.0001$) from the GFP control. Source data are provided as a Source Data file.

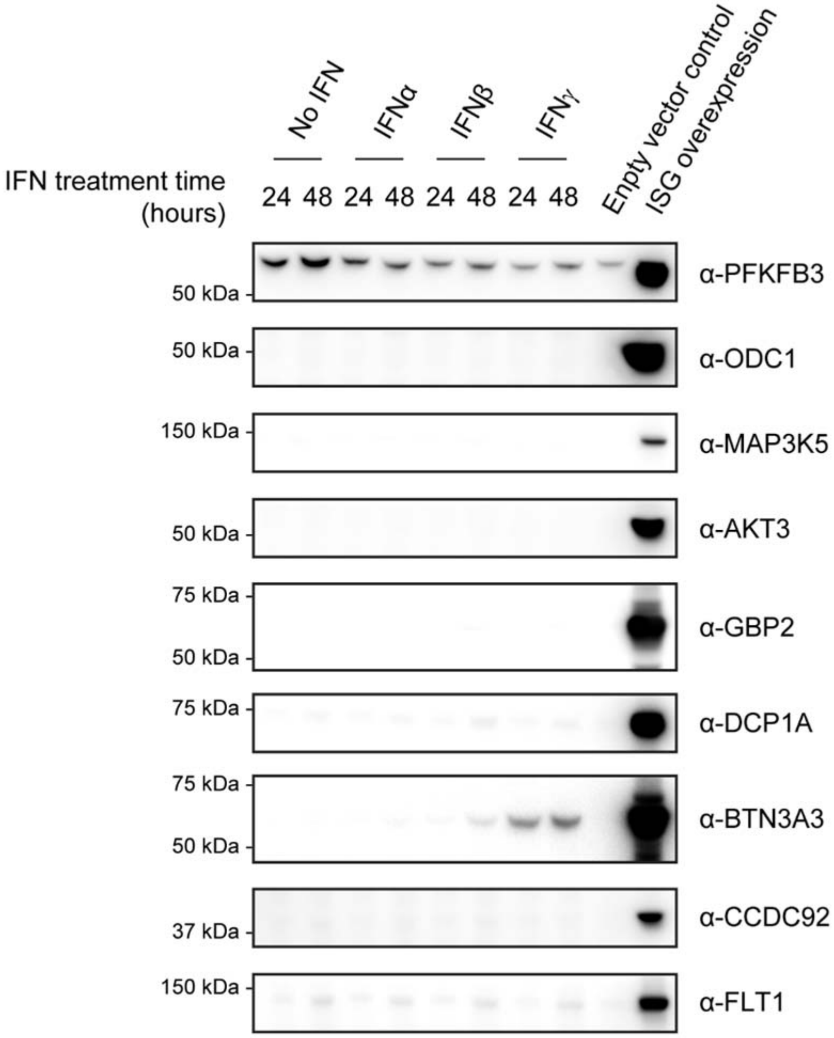

Supplementary Figure 4. ISG protein expression in HeLa cells treated with IFNs. HeLa cells were treated with or without 1000 unit ml^{-1} of IFNα, IFNβ, or IFNγ. After 24 or 48 h of treatment, cell lysates were collected and subjected to western blot analysis using the indicated ISG-specific antibodies. As a positive or negative control, cell lysates from HEK-293T cells transfected with vectors expressing the corresponded gene or its empty control vector were used, respectively. IFI6 protein expression was not assessed due to the lack of a commercially available antibody for IFI6 protein. Data are representative of two independent experiments. Source data are provided as a Source Data file.

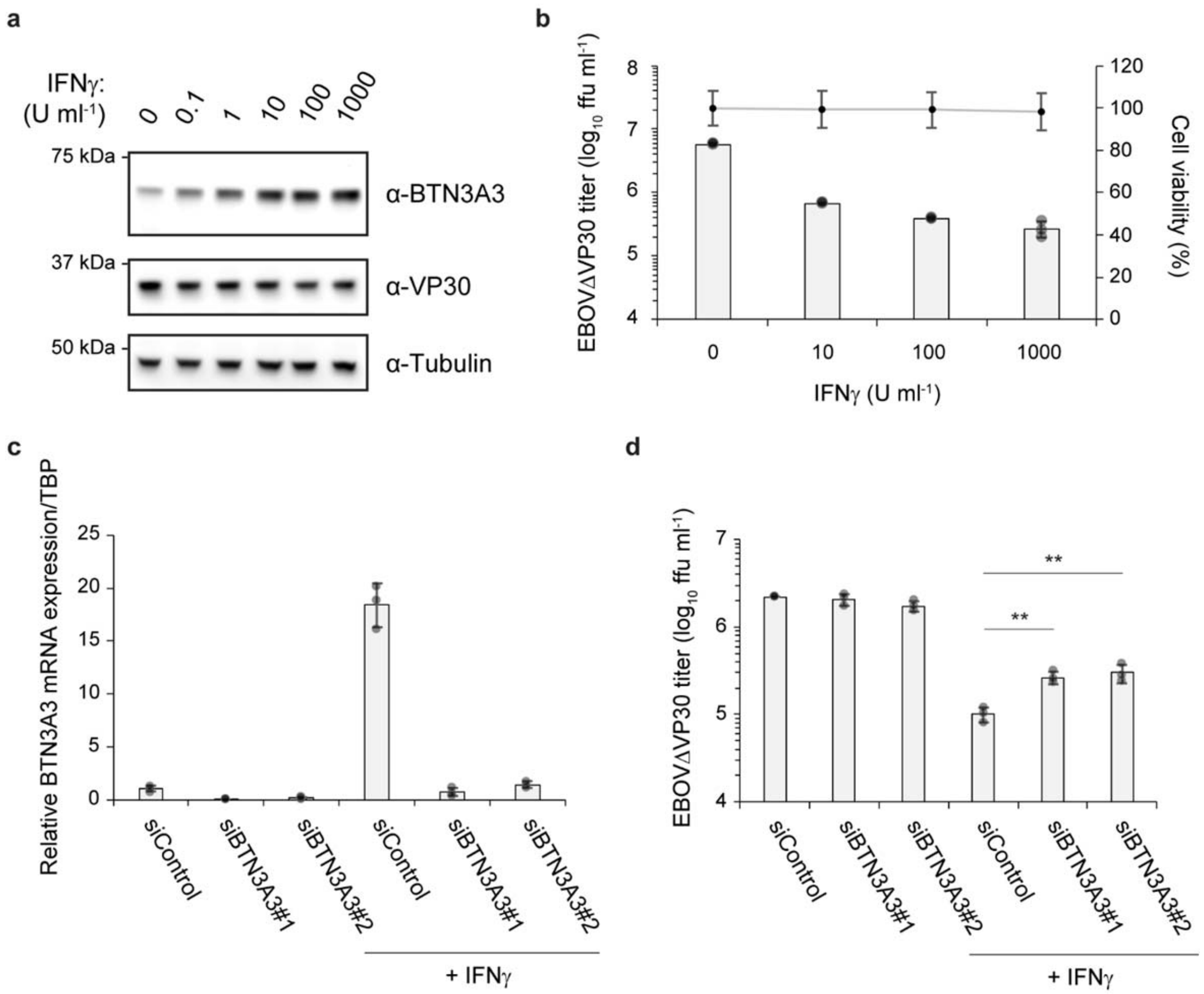

Supplementary Figure 5. EBOV attenuation by endogenous BTN3A3. (**a**) BTN3A3 expression in Huh7.0 VP30 cells treated with increasing doses of IFNγ for 24 h. Cells were lysed and the indicated protein expression levels were analyzed by immunoblotting. Data are representative of two independent experiments. (**b**) Titers of EBOVΔVP30 from Huh7.0 VP30 cells treated with increasing doses of IFNγ for 24 h prior to infection at an MOI of 0.01. Virus titer was measured on day 2 post-infection. In a separate set of experiments, cell viability of IFNγ-treated cells was measured on day 3 post-treatment by using a cell proliferation assay. Data are presented as means ± SD, and are representative of three independent experiments. (**c**) Relative BTN3A3 mRNA expression in Huh7.0 VP30 cells under BTN3A3 knockdown. Cells were transfected with BTN3A3 siRNAs or control siRNA and then at 48 h post-transfection, cells were treated with 100 U ml^{-1} of IFNγ for 24 h. RNA was quantified by qRT-PCR. Data are representative of two independent experiments performed in triplicate, and are presented as means ± SD of technical triplicates. (**d**) Cells were transfected with siRNA and then treated with IFNγ as described in (**c**). At 24 h post-IFNγ treatment, cells were infected with EBOVΔVP30 at an MOI of 0.01. Virus titer was measured on day 2 post-infection. Data are presented as means ± SD, and are representative of three independent experiments. (*) indicates a statistically significant difference (*p* values of two-tailed Student's *t*-tests; $^{**}p < 0.01$) from the control. Source data are provided as a Source Data file.

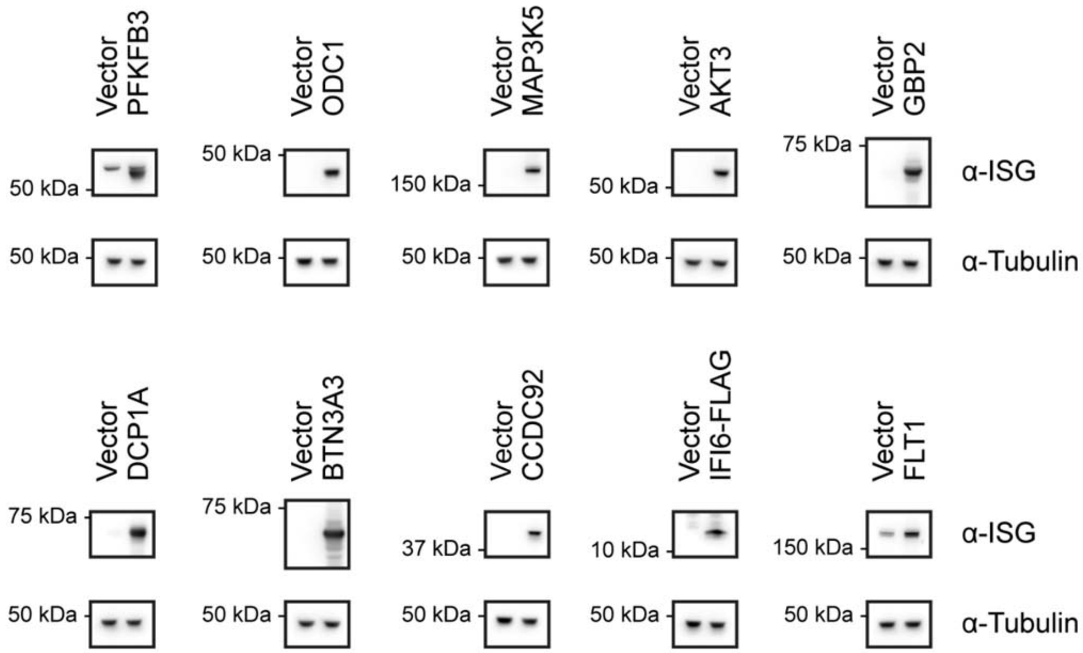

Supplementary Figure 6. ISG expression in the virus entry assay. HEK-293T cells were transfected with vectors expressing the indicated genes. At 24 h post-transfection, cell lysates were collected and subjected to western blot analysis. Source data are provided as a Source Data file.

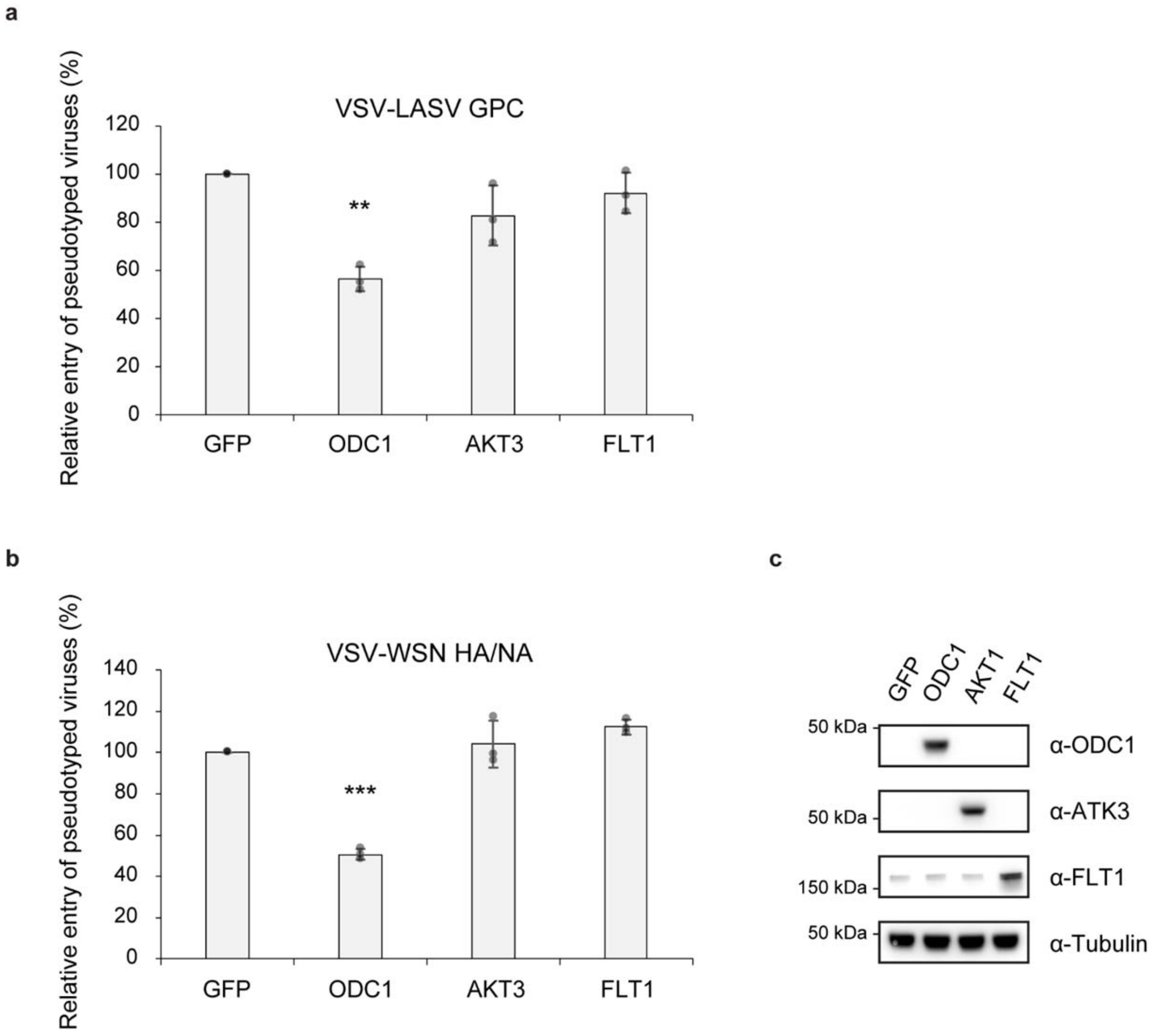

Supplementary Figure 7. Effect of three ISGs on Lassa and influenza virus entry. HEK-293T cells were transfected with vectors expressing the indicated genes for 24 h prior to infection with (**a**) VSV-LASV GPC or (**b**) VSV-WSN HA/NA. Data are presented as means ± SD, and are representative of three independent experiments. (*) indicates a statistically significant difference (*p* values of two-tailed Student's *t*-tests; $^{**}p < 0.01$, $^{***}p < 0.001$). (**c**) Expression of each ISG before infection was confirmed by western blot analysis. Source data are provided as a Source Data file.

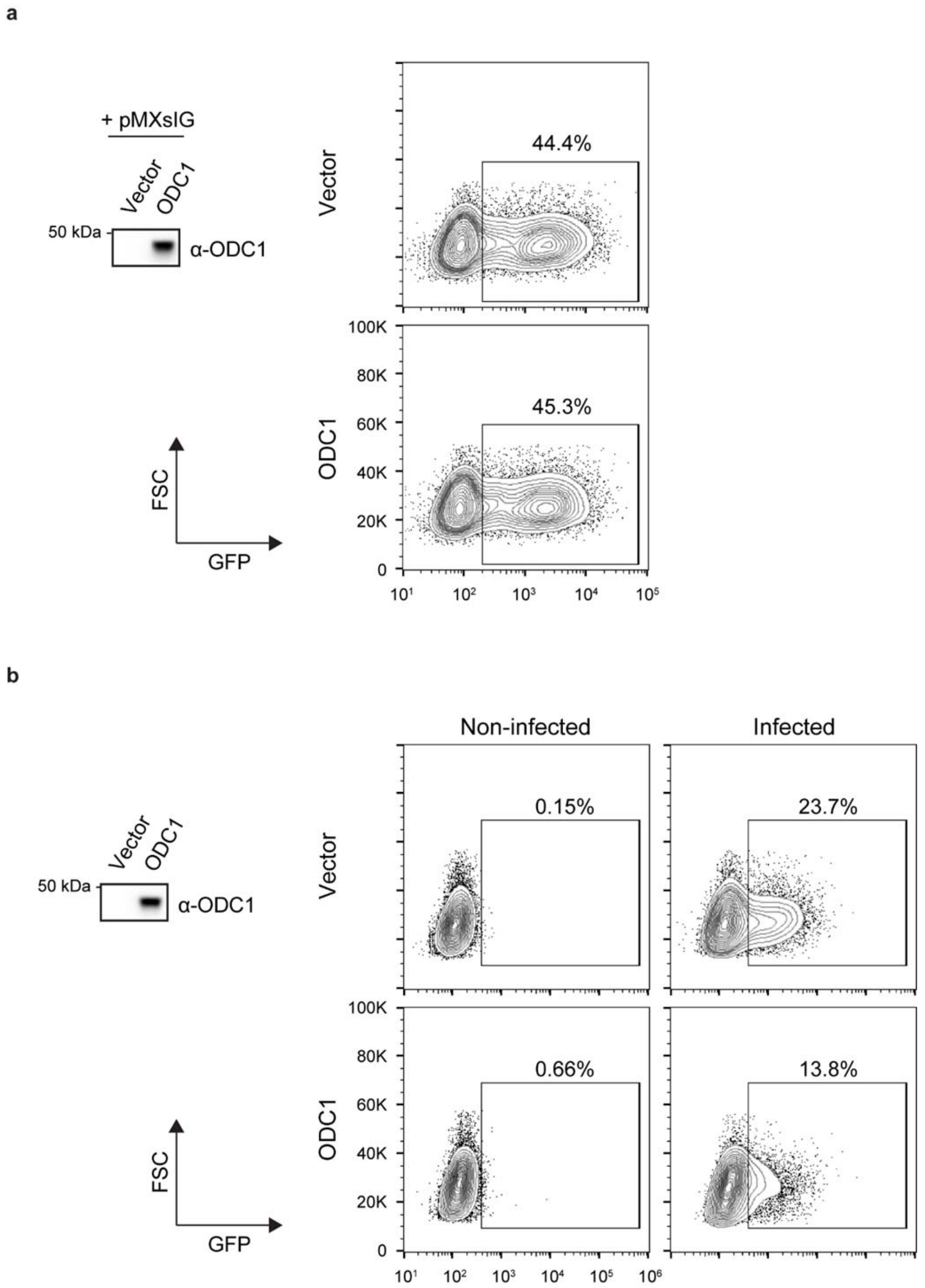

Supplementary Figure 8. Effect of ODC1 on VSV G-mediated retrovirus entry. (**a**) HEK-293T cells were transfected with a vector expressing ODC1 or an empty control vector together with the pMXs-IG retroviral vector. At 24 h post-transfection, GFP expression and ODC1 expression were analyzed by flow cytometry and western blot analysis, respectively. (**b**) HEK-293T cells were transfected with a vector expressing ODC1 or an empty control vector for 24 h prior to infection with a retrovirus bearing VSV G. At 24 h post-infection, GFP expression and ODC1 expression were analyzed by flow cytometry and western blot analysis, respectively. Data are representative of two independent experiments performed in triplicate. Source data are provided as a Source Data file.

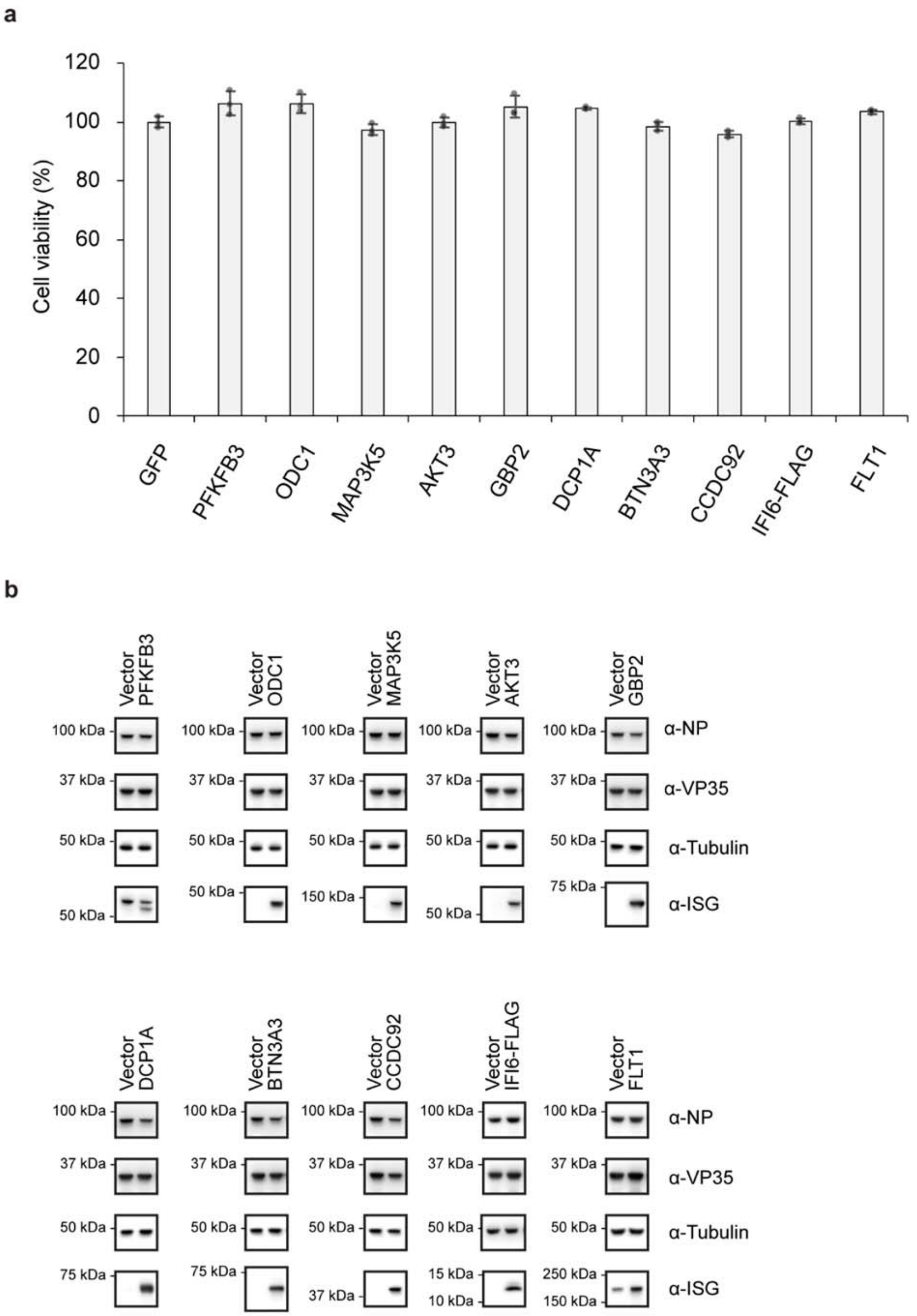

Supplementary Figure 9. ISG and viral protein expression in the EBOV minireplicon assay. HEK-293T VP30/L cells were transfected as described in Figure 3b. At 48 h post-transfection, cell lysates were collected and subjected to a (**a**) cell proliferation assay and (**b**) western blot analysis. Data are presented as means ± SD, and are representative of three independent experiments. Source data are provided as a Source Data file.

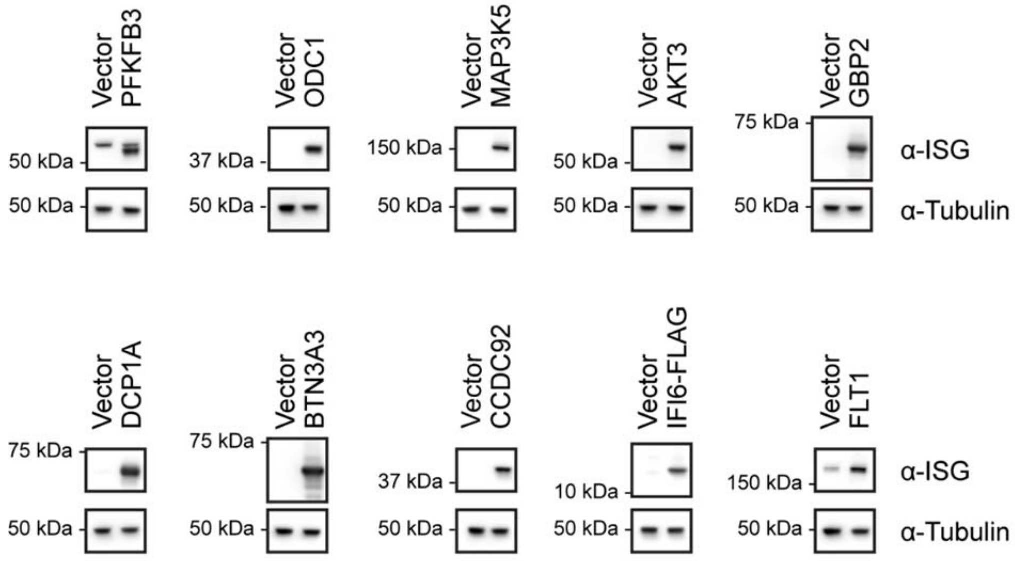

Supplementary Figure 10. ISG expression in the EBOV virion formation and budding assay. HEK-293T cells were transfected with vectors expressing the indicated genes as described in Figure 3c and d. At 24 h post-transfection, cell lysates were collected and subjected to western blot analysis. Source data are provided as a Source Data file.

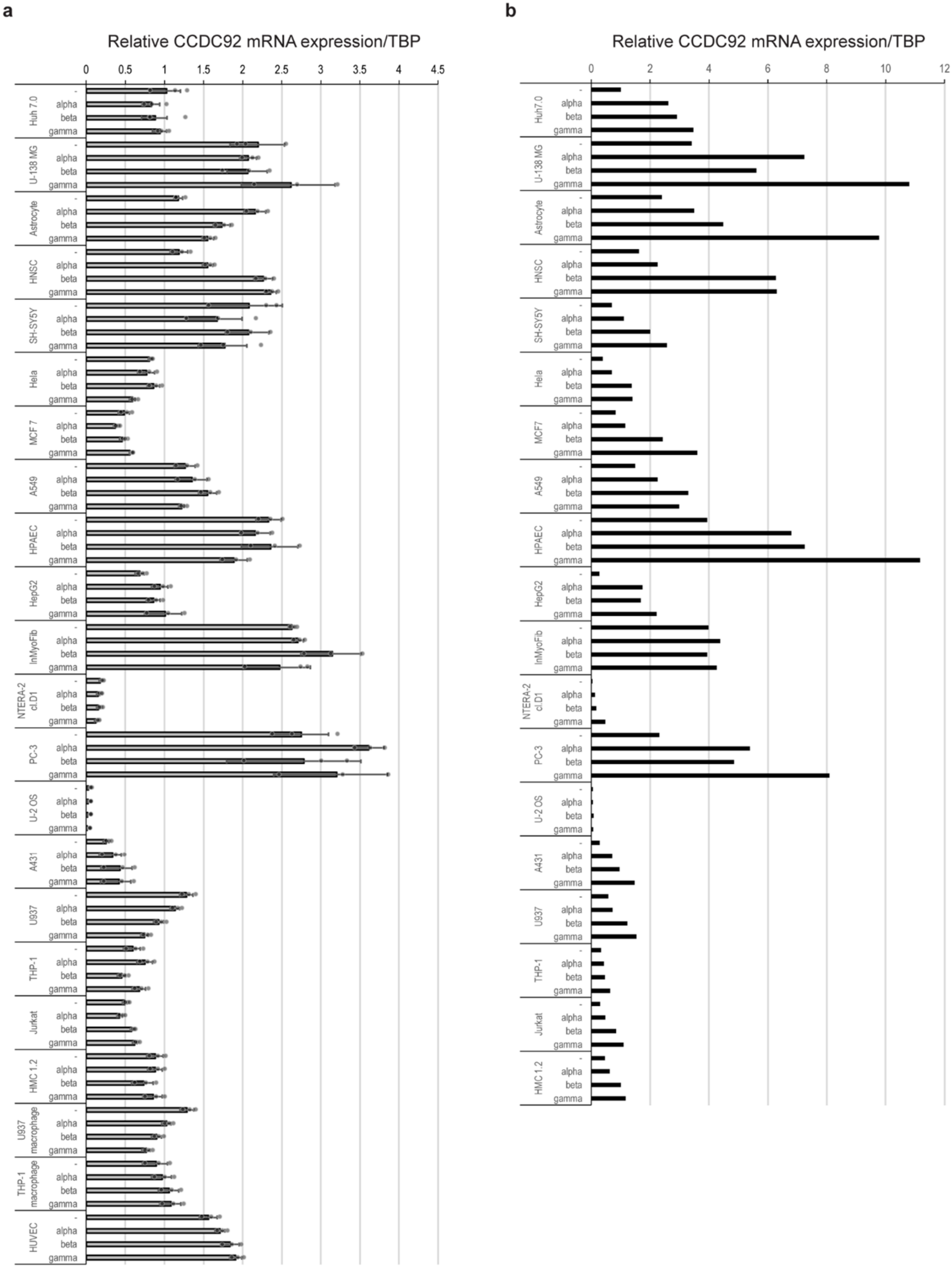

a
Relative CCDC92 mRNA expression/TBP
0 0.5 1 1.5 2 2.5 3 3.5 4 4.5
Huh 7.0
U-138 MG
Astrocyte
HNSC
SH-SY5Y
Hela
MCF 7
A549
HPAEC
HepG2
lnMyoFib
NTERA-2 cl.D1
PC-3
U-2 OS
A431
U937
THP-1
Jurkat
HMC 1.2
U937 macrophage
THP-1 macrophage
HUVEC
-
alpha
beta
gamma
b
Relative CCDC92 mRNA expression/TBP
0 2 4 6 8 10 12
Huh7.0
U-138 MG
Astrocyte
HNSC
SH-SY5Y
Hela
MCF7
A549
HPAEC
HepG2
lnMyoFib
NTERA-2 cl.D1
PC-3
U-2 OS
A431
U937
THP-1
Jurkat
HMC 1.2
-
alpha
beta
gamma

Supplementary Figure 11. Relative CCDC92 mRNA expression in cells treated with IFNs. Various cell lines and primary cells were treated with 1000 unit ml^{-1} of IFNα, IFNβ, or IFNγ for 24 h. Total cellular RNA was collected and analyzed as described in the Supplementary Methods. The results were obtained using two different systems: (**a**) the QuantStudio 6 Flex system and (**b**) the 7900ht Fast Real-time PCR system. The relative CCDC92 mRNA expression level was determined by setting the value of IFN-untreated Huh7.0 cells to 1.0. The mean of each experiment performed in (**a**) triplicate or (**b**) duplicate is shown. Data in (**a**) are presented as means ± SD. Source data are provided as a Source Data file.

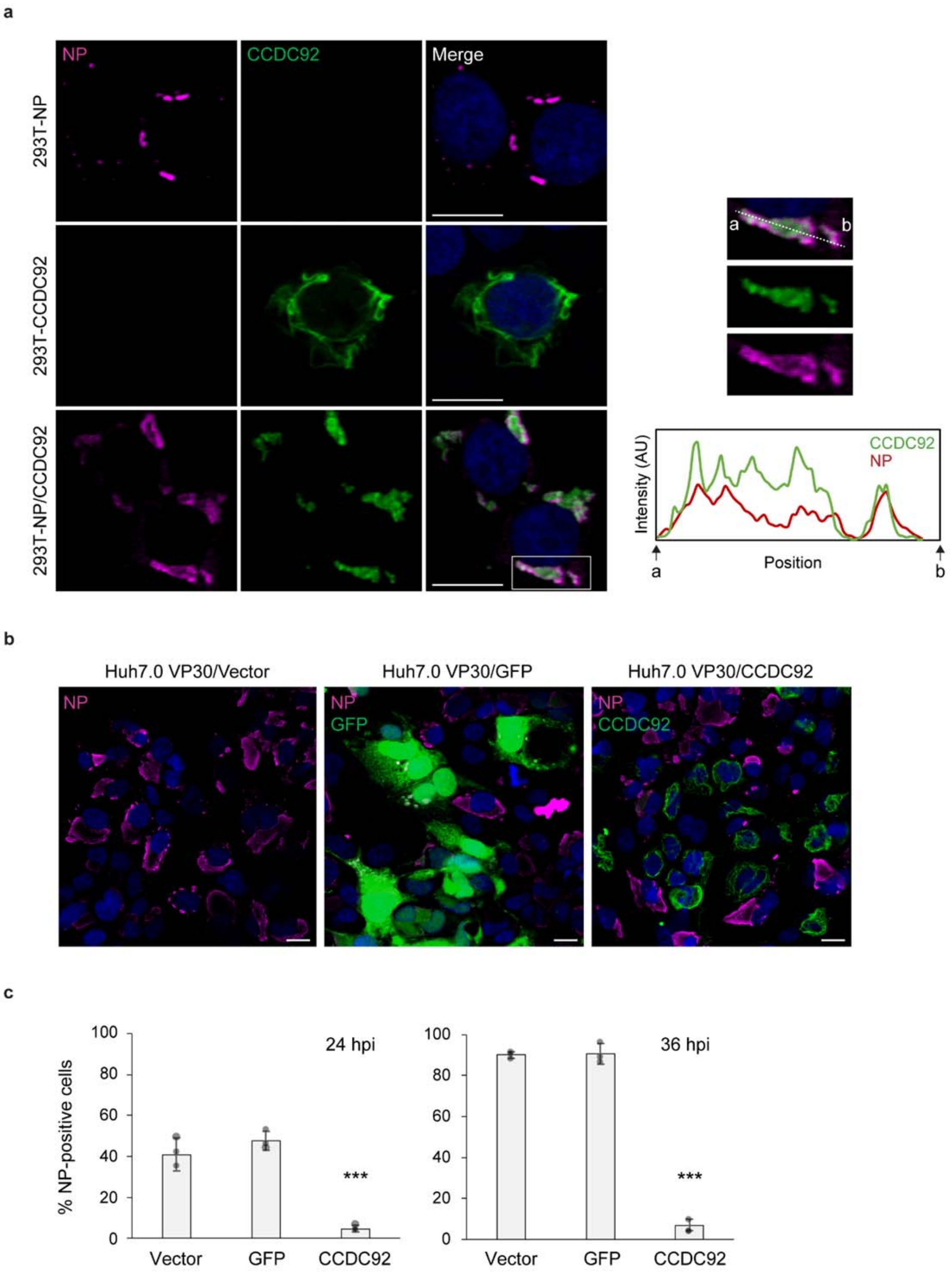
a
NP
CCDC92
Merge
293T-NP
293T-CCDC92
293T-NP/CCDC92
a
b
CCDC92
NP
Intensity (AU)
Position
a
b
b
Huh7.0 VP30/Vector
Huh7.0 VP30/GFP
Huh7.0 VP30/CCDC92
NP
NP
GFP
NP
CCDC92
c
% NP-positive cells
24 hpi
36 hpi
100
80
60
40
20
0
***
***
Vector
GFP
CCDC92
Vector
GFP
CCDC92

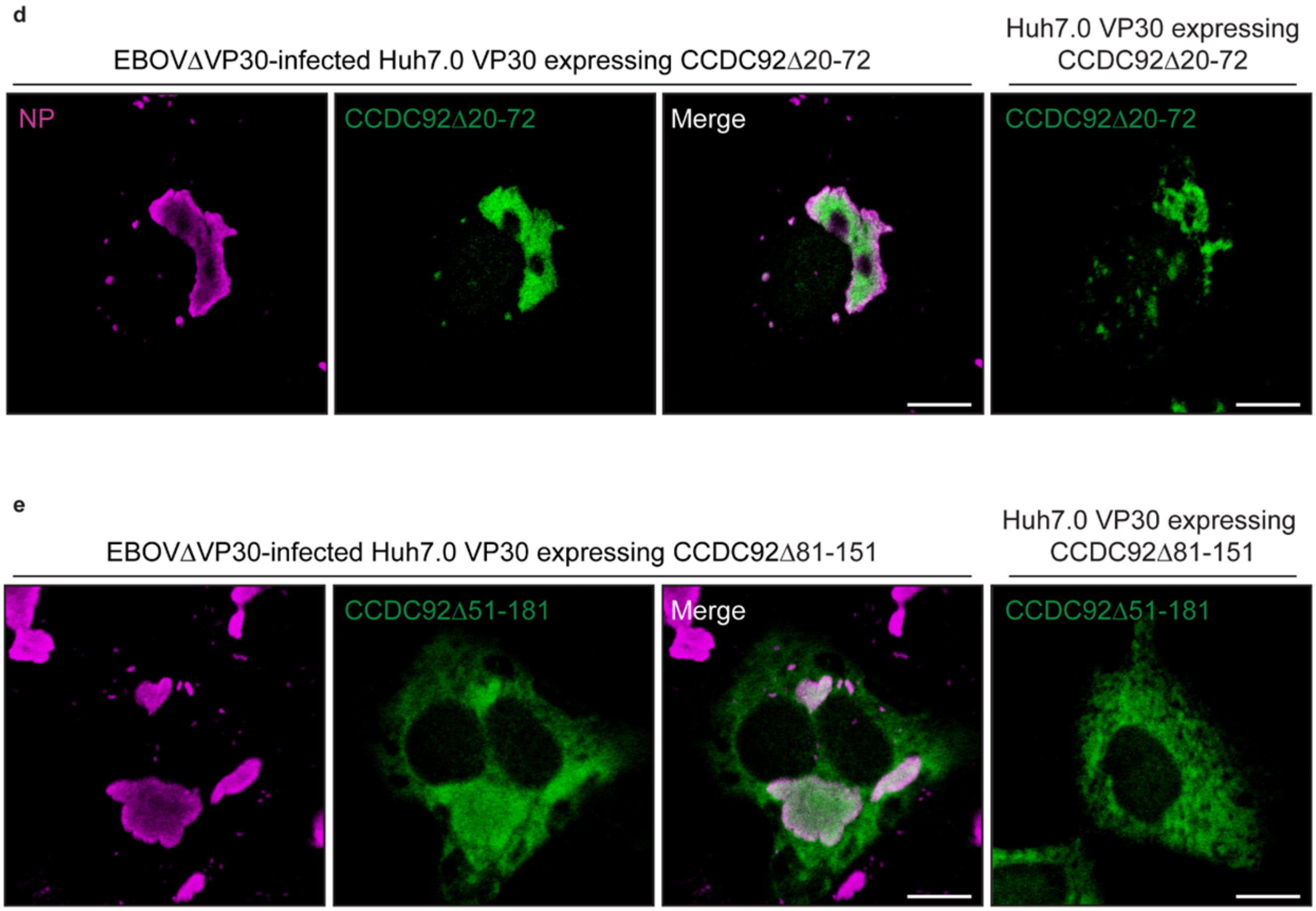

Supplementary Figure 12. Localization of NP and CCDC92 in transfected or infected cells. (**a**) EBOV NP (magenta) and/or CCDC92 (green) in HEK-293T cells transfected with vectors expressing NP with or without CCDC92 were visualized with specific antibodies and analyzed by confocal microscopy. Nuclei of cells were visualized with DAPI (blue). Enlarged images corresponding to the boxed areas are shown next to the original images. The white dashed line indicates the quantified region. The line profile plots indicate relative fluorescence intensity in arbitrary units (AU) of CCDC92 and NP. Scale bars, 20 µm. (**b**) EBOV NP expression (magenta) in Huh7.0 VP30 cells after overexpression of CCDC92 or GFP (green). Cells were transfected with vectors expressing CCDC92 or GFP, or an empty control vector for 24 h prior to infection with EBOVΔVP30 at an MOI of 3.0. At 36 h post-infection, the expression of the indicated proteins was visualized with specific antibodies and analyzed by confocal microscopy. Cell nuclei were visualized with DAPI (blue). Scale bars, 20 µm. (**c**) Huh7.0 VP30 cells were transfected and infected as described in (**b**). At 24 and 36 h post-infection, the number of infected cells (NP-positive cells) was counted. The ratio of double-positive cells (NP/CCDC92-positive cells or NP/GFP-positive cells) to total CCDC92-positive or GFP-positive cells is presented as a percentage. For the empty vector control, the ratio of NP-positive cells to total cells is presented. At least 100 cells were counted in each experiment, performed three times. Data are presented as the mean ± SD. (*) indicates a statistically significant difference (*p* values of two-tailed Student's *t*-tests; ***$p < 0.001$) from the GFP control. (**d and e**) Localization of NP and CCDC92 deletion mutants in EBOVΔVP30-infected cells. EBOV NP (magenta) and/or CCDC92 (green) in Huh7.0 VP30 cells infected with EBOVΔVP30 24 h prior to transfection with expression vectors for either CCDC92 (**d**) Δ20-72 or (**e**) Δ81-151 were visualized with specific antibodies and analyzed by confocal microscopy. The panels to the right represent each CCDC92 deletion mutant (green) in transfected cells without virus infection. Scale bars, 20 µm. Data (**a**, **d**, and **e**) are representative of two independent experiments. Source data are provided as a Source Data file.

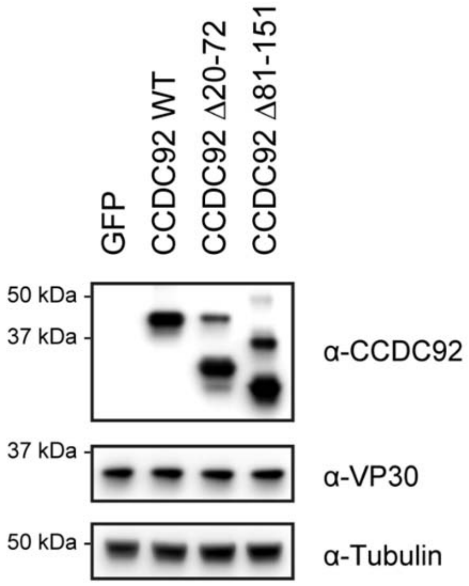

Supplementary Figure 13. Effect of CCDC92 deletion mutants on EBOV-driven luciferase activity. HEK-293T VP30 cells were transfected with vectors expressing the indicated genes as described in Figure 4e. At 24 h post-transfection, cell lysates were collected and subjected to western blot analysis. Source data are provided as a Source Data file.

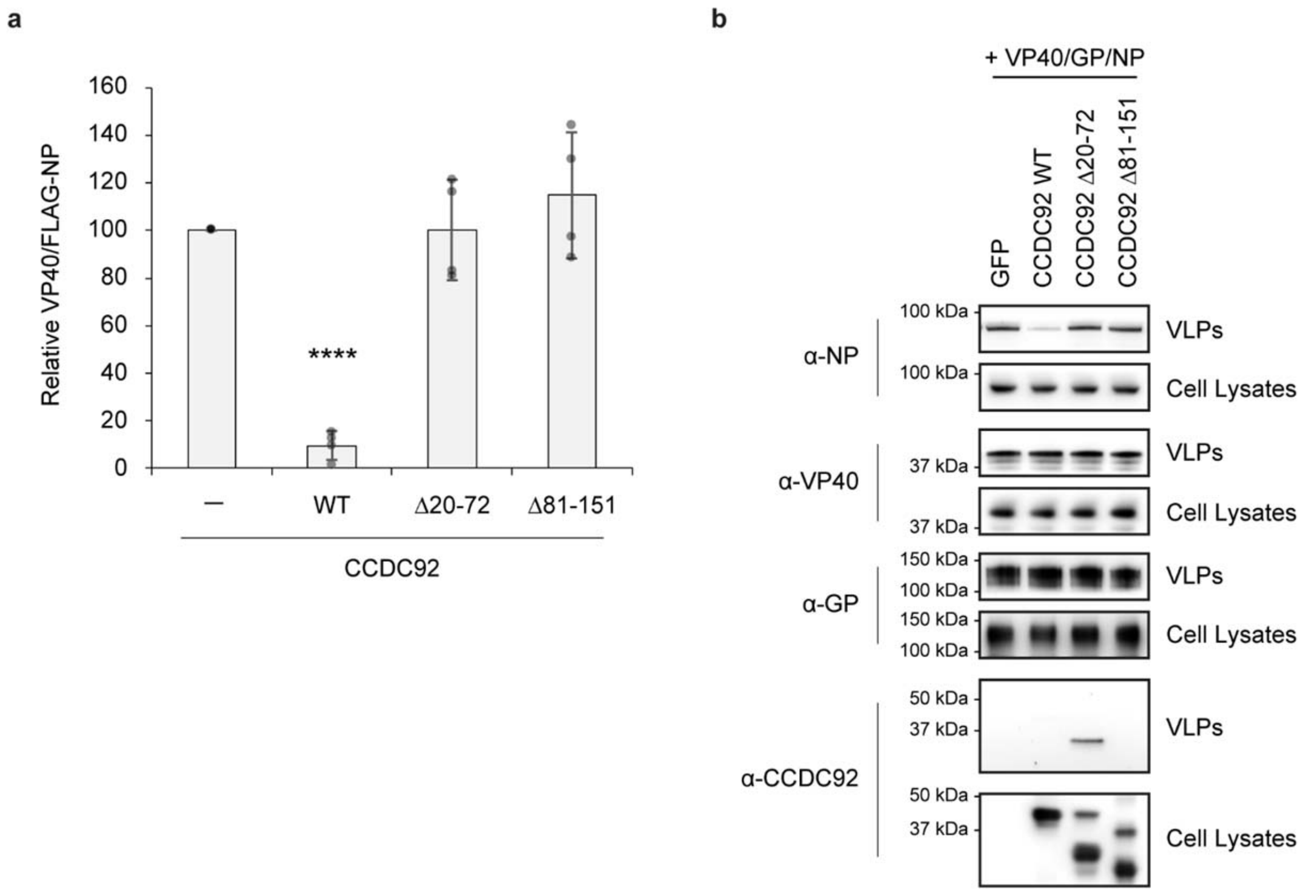

Supplementary Figure 14. Effect of CCDC92 deletion mutants on the NP–VP40 interaction and EBOV virion formation/budding. (**a**) Relative band intensity of VP40 precipitated with NP in the presence of CCDC92 or its deletion mutants in HEK-293T cells transfected as described in Figure 5b. Quantification analysis was performed using ImageJ software. The relative band intensities of precipitated VP40 were normalized against those of precipitated FLAG-NP. Data are presented as percentages ± SD ($n = 4$). (*) indicates a statistically significant difference (*p* value of a two-tailed Student's *t*-test; ****$p < 0.0001$) from the control. (**b**) HEK-293T cells were transfected with vectors expressing VP40, NP, GP, and the indicated CCDC92 constructs. At 48 h post-transfection, cell supernatants and cell lysates were collected and subjected to western blot analysis. Data are representative of four independent experiments. Source data are provided as a Source Data file.

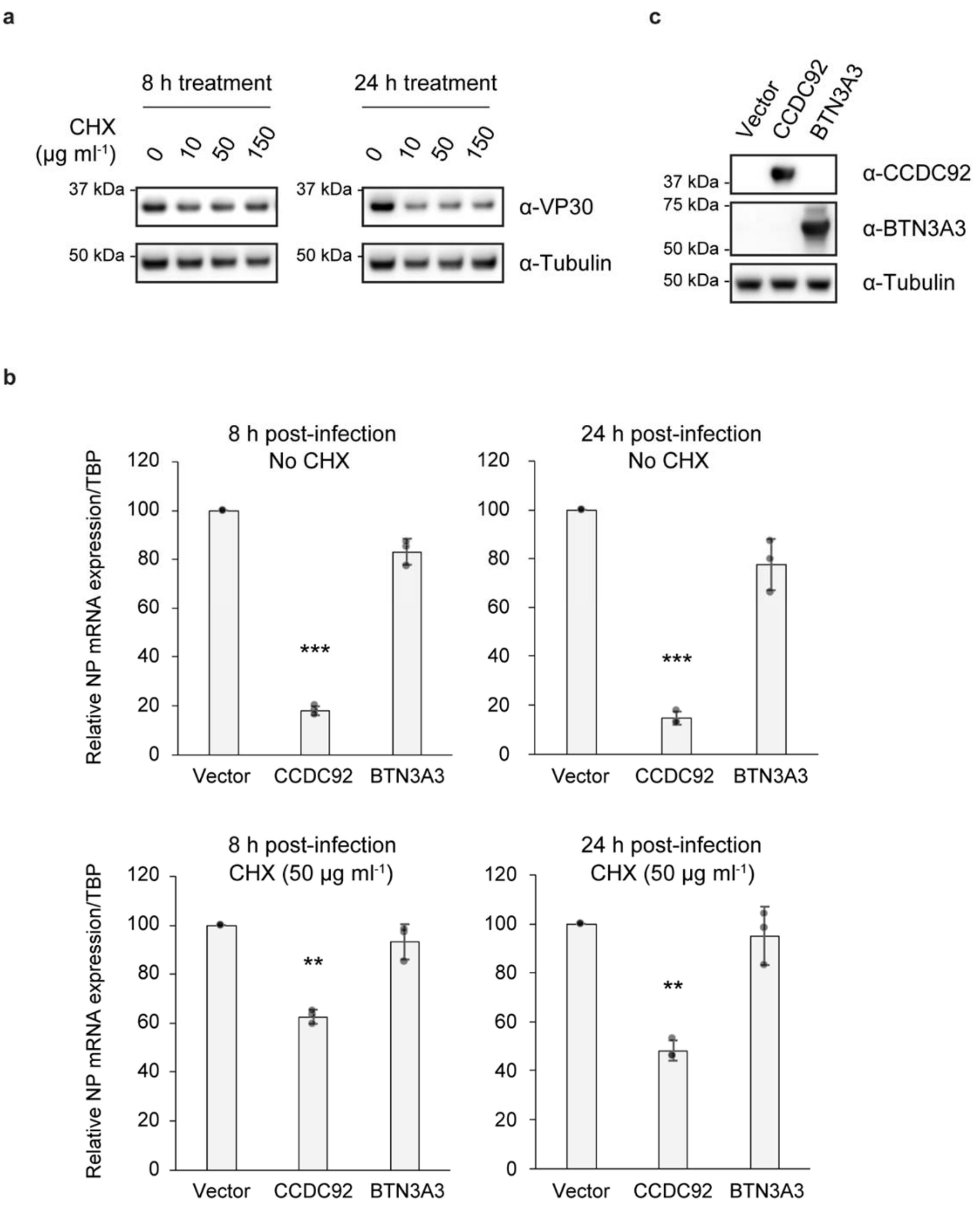

Supplementary Figure 15. Inhibition of NP mRNA expression by CCDC92 under CHX treatment. (**a**) HEK-293T VP30 cells were treated with CHX at 10–150 μg ml^{-1} for 8–24 h. VP30 expression was analyzed by western blot analysis. Data are representative of two independent experiments. (**b**) HEK-293T VP30 cells transfected for 24 h with vectors expressing the indicated genes were infected with EBOV∆VP30 in the presence or absence of CHX (50 μg ml^{-1}). At 8 or 24 h post-infection, RNA was extracted and then quantified by qRT-PCR. The results are normalized to TBP mRNA expression, and the data are presented as percentages ± SD (n = 3). (*) indicates a statistically significant difference (*p* values of two-tailed Student's *t*-tests; **$p < 0.01$ ***$p < 0.001$) from the control. (**c**) Expression of each ISG before CHX treatment was confirmed by western blot analysis. Source data are provided as a Source Data file.

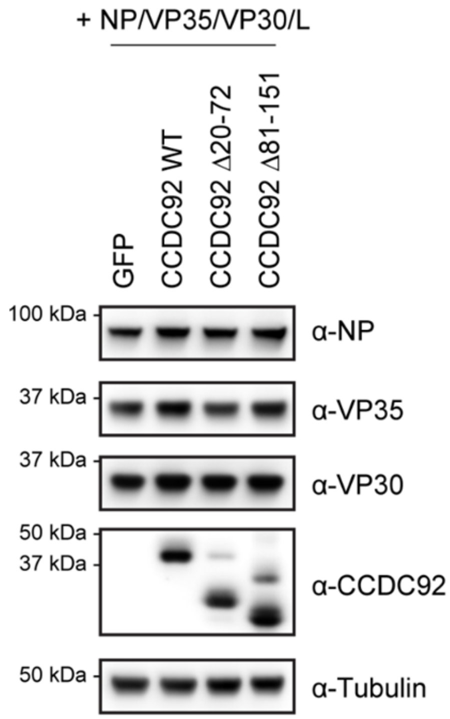

Supplementary Figure 16. Expression of CCDC92 deletion mutants in an EBOV minireplicon system. HEK-293T VP30 cells were transfected with vectors expressing the remaining minireplicon components, the indicated genes, and an internal Renilla luciferase control vector as described in Figure 5e. At 48 h post-transfection, cell lysates were collected and subjected to western blot analysis. Source data are provided as a Source Data file.

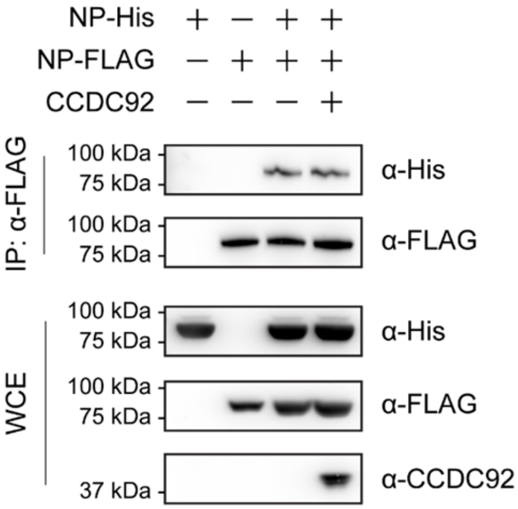

Supplementary Figure 17. Effect of CCDC92 on NP oligomerization. HEK-293T cells were transfected with the indicated combination of vectors. Cell lysates were immunoprecipitated with anti-FLAG antibody followed

by immunoblotting. Data are representative of three independent experiments. IP, immunoprecipitation. WCE, whole-cell extract. Source data are provided as a Source Data file.

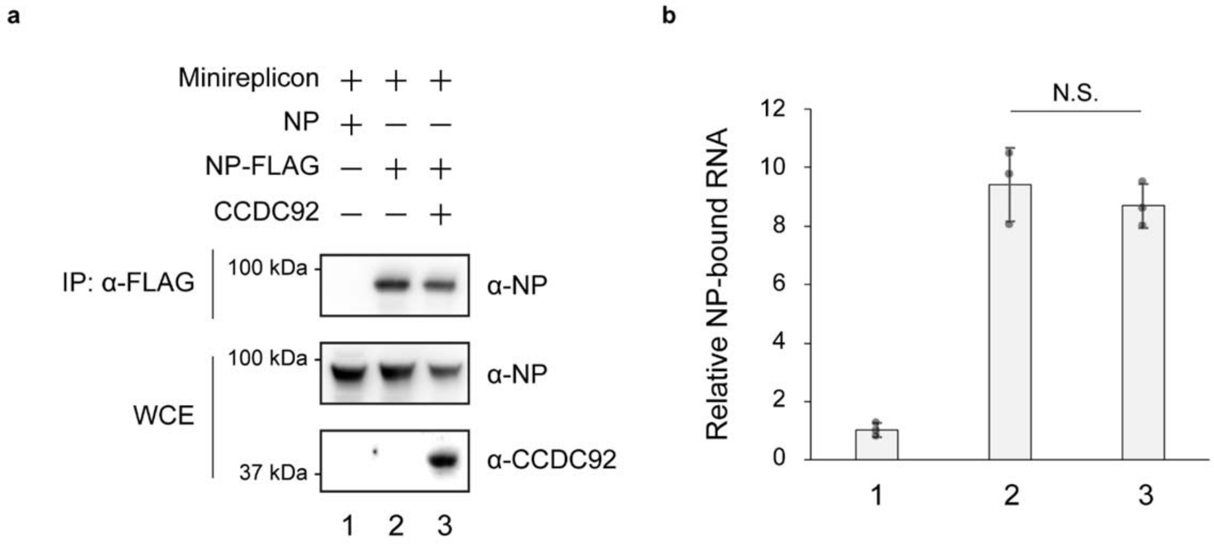

Supplementary Figure 18. Effect of CCDC92 on NP–RNA binding. HEK-293T cells were transfected with the indicated combination of vectors. (**a**) Cell lysates were immunoprecipitated with anti-FLAG antibody followed by immunoblotting. (**b**) Quantification of EBOV minireplicon RNA was performed by use of qRT-PCR. The amount of EBOV minireplicon RNA precipitated with the FLAG-tagged NP was normalized against the amount of EBOV minireplicon RNA in the whole cell lysates for each sample. Data are representative of three independent experiments performed in triplicate. The numbers under the blots correspond to the numbers in (**b**). IP, immunoprecipitation. WCE, whole-cell extract. N.S., not significant in a two-tailed Student's *t*-test. Source data are provided as a Source Data file.

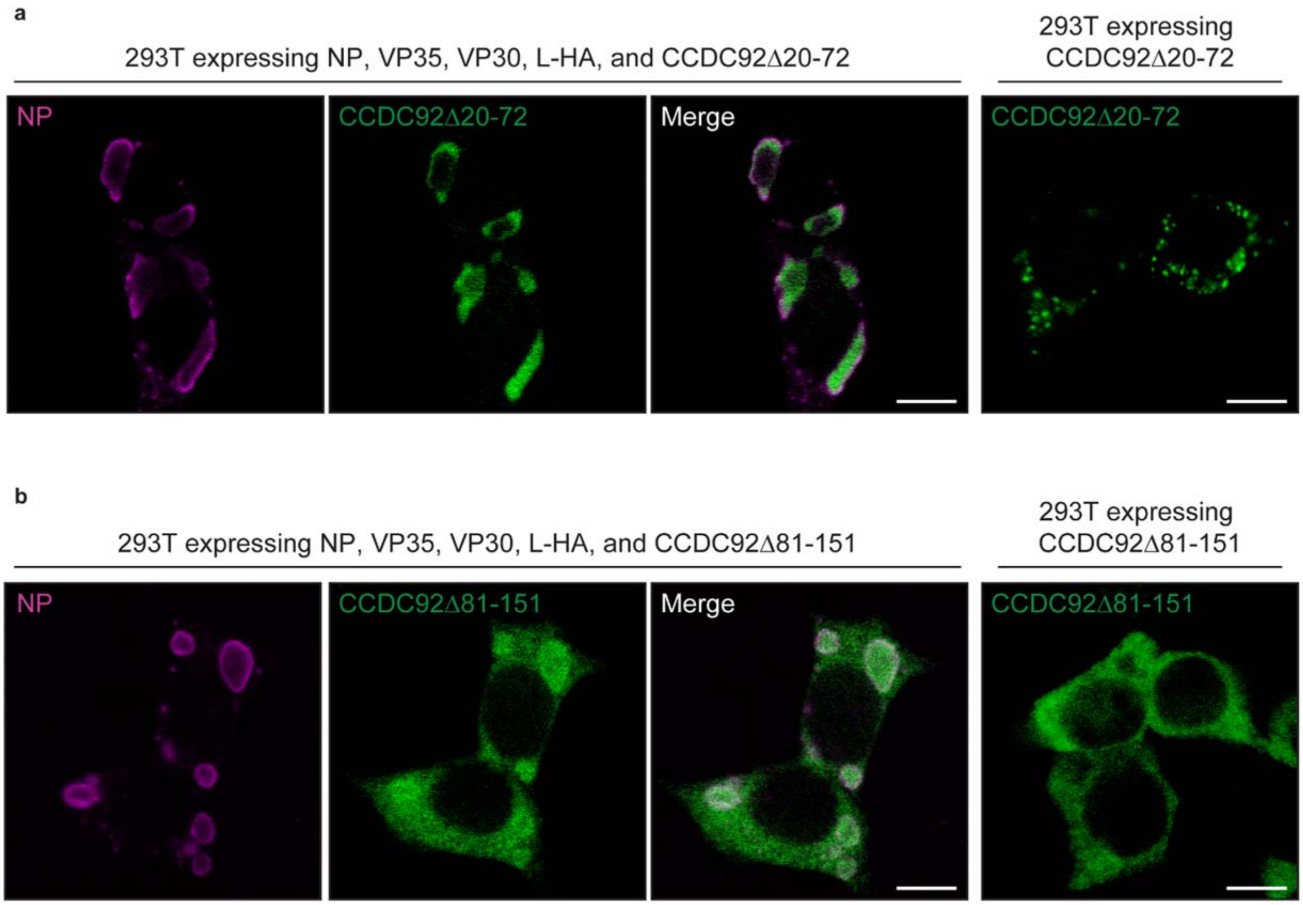

Supplementary Figure 19. Localization of NP and CCDC92 deletion mutants in cells forming viral inclusion bodies. EBOV NP (magenta) and CCDC92 (green) in HEK-293T cells transfected with vectors expressing EBOV proteins (NP, VP35, VP30, and L-HA) together with either CCDC92 (**a**) Δ20-72 or (**b**) Δ81-151 were visualized with specific antibodies and analyzed by confocal microscopy. The panels to the right represent CCDC92 (green) in cells transfected with a vector expressing each CCDC92 alone. Data are representative of two independent experiments. Scale bars, 20 μm.

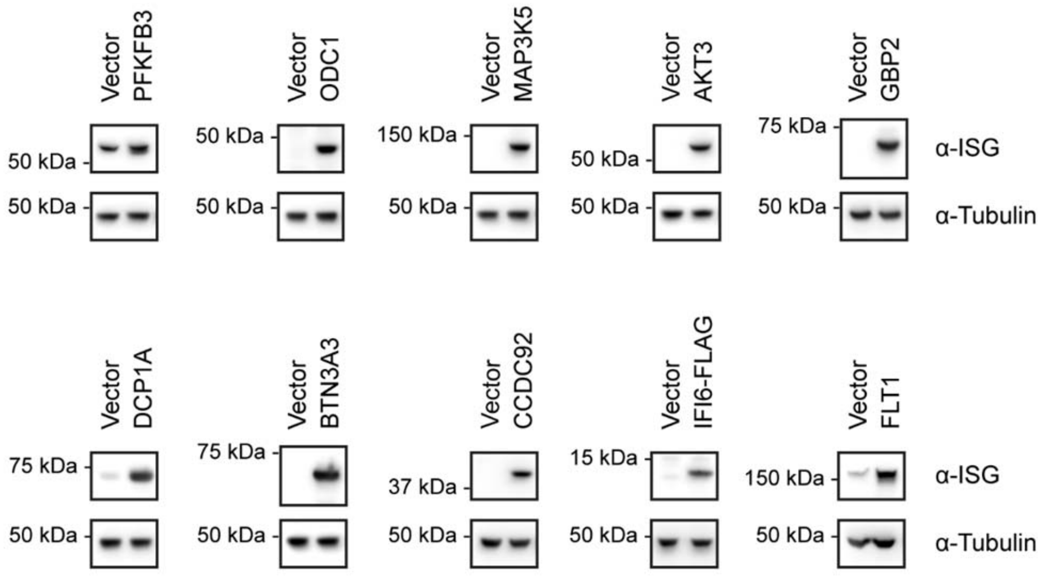

Supplementary Figure 20. ISG expression under the primary ISG screen conditions. HEK-293T VP30 cells were transfected with the indicated LentiX vectors. At 24 h post-transfection, cell lysates were collected and subjected to western blot analysis. Source data are provided as a Source Data file.

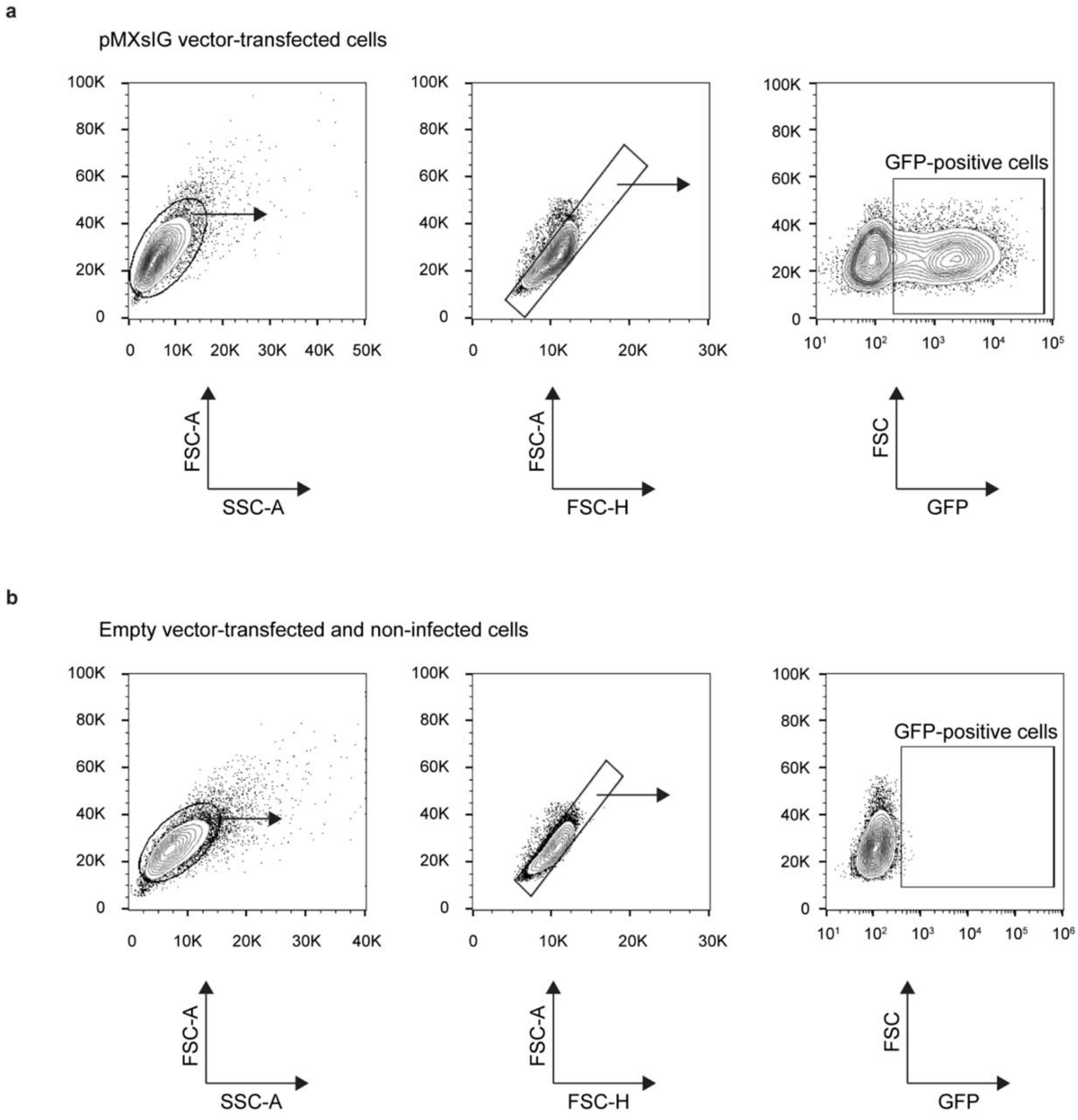

Supplementary Figure 21. Gating strategy. Gating strategy for flow cytometric analysis of GFP expression in transfected cells (**a**) and infected cells (**b**) presented in Supplementary Figures 8a and 8b, respectively. The same strategy was used for the analysis of GFP expression in the other cells presented in Supplementary Figures 8a and 8b.

Supplementary Tables 1-3

Supplementary Table 1: Selected interferon-stimulated genes (ISGs) that attenuate EBOV

Gene	Full Name	Target step(s) in the EBOV life cycle
PFKFB3	6-Phosphofructo-2-Kinase/Fructose-2,6-Biphosphatase 3	Replication/transcription
ODC1	Ornithine Decarboxylase 1	Entry
MAP3K5	Mitogen-Activated Protein Kinase Kinase Kinase 5	Replication/transcription
AKT3	V-Akt Murine Thymoma Viral Oncogene Homolog 3	Entry
GBP2	Guanylate Binding Protein 2, Interferon-Inducible	Replication/transcription
DCP1A	Decapping MRNA 1A	Replication/transcription
BTN3A3	Butyrophilin, Subfamily 3, Member A3	Virion formation/budding
CCDC92	Coiled-Coil Domain Containing 92	Replication/transcription and virion formation/budding
IFI6	Interferon, Alpha-Inducible Protein 6	Replication/transcription
FLT1	Fms-Related Tyrosine Kinase 1	Entry

Supplementary Table 2: Fold changes in ISG mRNA expression after IFN treatment (1000 U ml^{-1} for 24 h)

ISG	IFNα/TBP		IFNβ/TBP		IFNγ/TBP	
	HeLa	Huh7.0	HeLa	Huh7.0	HeLa	Huh7.0
**PFKFB3**	0.9	1.1	0.9	2.3	0.8	0.8
**ODC1**	0.7	1.0	0.5	1.0	0.6	0.7
**MAP3K5**	1.1	0.9	1.3	1.1	0.9	0.8
**AKT3**	1.4	1.6	3.8	2.0	1.4	0.9
**GBP2**	2.1	1.9	6.2	10.4	21.0	20.8
**DCP1A**	1.7	1.1	3.4	2.0	1.2	1.1
**BTN3A3**	1.8	4.5	4.2	19.3	9.3	17.9
**CCDC92**	1.1	1.0	1.2	1.0	0.9	0.8
**IFI6**	547.5	2281.0	1056.3	3721.0	30.8	203.5
**FLT1**	n.d.	n.d.	n.d.	n.d.	n.d.	n.d.

n.d.: non-detactable level of mRNA. Source data are provided as a Source Data file.

Supplementary Table 3: Cell list used in Supplementary Fig. 11

Cells described in Supplementary Fig. 11		Culture media
Huh7.0	Human hepatocarcinoma cell line (Laboratory stock)	DMEM (12-614Q, Lonza), 2 mM L-glutamine, 10% FBS
U-138 MG	Human brain glioblastoma (HTB-16, ATCC)	EMEM (30-2003, ATCC), 10% FBS
Astrocyte	Normal Human Astrocytes (NHA) (CC-2565, Lonza)	Human Astrocyte Medium Kit (821K-500, Cell Applications)
HNSC	Human Neural Stem Cells (H9-Derived) (N7800-200, Thermo)	KnockOut DMEM/F-12, StemPro Neural Supplement, 20 ng ml^{-1} bFGF, 20 ng ml^{-1} EGF, 2 mM GlutaMAX
SH-SY5Y	Huamn bone marrow neuroblastoma (CRL-2266, ATCC)	1:1 mixture of EMEM (30-2003, ATCC) and F12 Ham (N4888, Sigma), 10% FBS
HeLa	Human cervical cancer cell line (Laboratory stock)	DMEM (12-614Q, Lonza), 2 mM L-glutamine, 10% FBS
MCF7	Human breast cancer cell line (HTB-22, ATCC)	EMEM (30-2003, ATCC), 0.01 mg ml^{-1} human recombinant insulin (91077C-100MG, Sigma)
A549	Human lung carcinoma cell line (Laboratory stock)	F-12K Medium (30-2004, ATCC), 10%FBS
HPAEC	Human Pulmonary Artery Endothelial cells (HPAEC) (CC-2530, Lonza)	Human Endothelial Cell Growth Medium Kit (211K-500, Cell Applications)
HepG2	Human hepatoma cell line (Laboratory stock)	DMEM (12-614Q, Lonza), 2 mM L-glutamine, 10% FBS
InMyoFib	Human Intestinal Myofibroblasts (H-InMyoFib) (CC-2902, Lonza)	Human Dermal Fibroblast Growth Medium Kit (116K-500a, Cell Applications)
NTERA-2 cl.D1	Human testicular cell line (CRL-1973, ATCC)	DMEM (30-2002, ATCC), 10% FBS
PC-3	Human prostatic cancer cell line (CRL-1435, ATCC)	F-12K Medium (30-2004, ATCC), 10% FBS
U-2 OS	Human osteosarcoma cell line (HTB-96, ATCC)	McCoy's 5a Medium (30-2007, ATCC), 10% FBS
A431	Human epidermoid carcinoma cell line (CRL-1555, ATCC)	DMEM (30-2002, ATCC), 10% FBS
U937	Human monocytic cell line (Laboratory stock)	RPMI 1640 (11875093, Life Technologies), 10% FBS
THP-1	Human monocytic cell line (Laboratory stock)	RPMI 1640 (11875093, Life Technologies), 0.05 mM 2-mercaptoethanol, 10% FBS
Jurkat	Human T leukemia (Laboratory stock)	RPMI 1640 (11875093, Life Technologies), 10% FBS
HMC 1.2	Human mast cell (SCC062, Millipore Sigma)	IMDM (#12440-053, Life Technologies), 1.2 mM alpha-thioglycerol (M6145, Sigma), 10% FBS
HUVEC	Human Umbilical Vein Endothelial Cells (HUVEC) (CC-2519, Lonza)	EGM BulletKit (CC-3124)

Supplementary Methods

Luciferase reporter assay

To examine the luciferase activity under ISG overexpression, HEK-293T cells were transfected with luciferase expression vectors (pCAGGS firefly luciferase [0.1 µg] and pCAGGS Renilla luciferase [0.002 µg]) together with the pCAGGS ISG expression vectors (0.4 µg each) by using TransIT-LT1 (Mirus). Twenty-four hours later, cells were lysed and subjected to the dual-luciferase assay by following the manufacturer's protocol (Promega).

Generation and Neon electroporation of HUVEC VP30 cells

HUVEC VP30 cells (human umbilical vein endothelial cells stably expressing EBOV VP30) were generated as follows: a cDNA fragment encoding EBOV VP30 was cloned into the MLV retroviral vector pMXs-IRES-Neo (pMXs-IN) (Cell Biolabs). To generate the retrovirus, Plat-GP cells (Cell Biolabs) were co-transfected with pMXs-IN encoding EBOV VP30 along with an expression vector for VSV G by using Lipofectamine 2000 (Invitrogen). Two days later, the culture supernatants containing the retroviruses were collected, clarified through 0.45-µm-pore filters, and then used to infect HUVEC cells. Stable cells were selected with 500 µg ml^{-1} G418 (InvivoGen).

DNA electroporation was performed with the Neon transfection system (Invitrogen) by following the manufacturer's instructions with slight modifications. Briefly, HUVEC VP30 cells at 4–6 passages (2.5×10^5 cells in 10 µl of resuspension buffer R [Neon transfection system 10 µl kit]) were mixed with 400 ng of vector and then treated with a single pulse of 1350 V for 30 ms in a 10-µl electroporation tip. Cells were seeded in 96-well tissue culture plates at 2×10^4 cells per well and at 24 h post-transfection, the cells were subjected to a cell viability assay, western blot analysis, and infection with EBOVΔVP30-luc.

Generation of VSV-LASV GPC and VSV-WSN HA/NA

Both VSV-LASV GPC and VSV-WSN HA/NA were prepared like VSV-EBOV GP. Briefly, HEK-293T cells were transfected for 24 h with pCAGGS protein expression vectors for LASV GPC or WSN HA and NA and then infected with parental VSV-G. At 24 h post-infection, supernatants containing viruses were collected and then used for the entry assay.

Flow cytometric analysis

HEK-293T cells co-transfected for 24 h with the MLV retroviral vector pMXs-IRES-GFP (Cell Biolabs) together with the expression vector for ODC1, as well as HEK-293T cells transfected for 24 h with the expression vector for ODC1 and then infected for 24 h with the retrovirus bearing VSV-G, were detached with 0.25% trypsin, washed with PBS, and fixed with 4% paraformaldehyde for 10 min at room temperature. These cells were then analyzed with a FACS Aria III flow cytometer (BD Biosciences) and FlowJo software (Tree Star) using the gating strategy shown in Supplementary Figure 21.

Quantitative reverse transcription PCR (qRT-PCR)

RNA was isolated with an RNeasy Mini Kit (Qiagen). Approximately 400–600 ng of total RNA from cells was transcribed into cDNA by using a QuantiTect Reverse Transcription Kit (Qiagen). The cDNA was amplified and analyzed by using a QuantiTect SYBR Green PCR Kit (Qiagen) on the 7900ht Fast Real-time PCR system (Applied Biosystems) or by using PowerUp SYBR Green Master Mix (Life Technologies) on QuantStudio 6 Flex (Applied Biosystems) following the manufacturer's protocol. Primers targeting ISGs and TATA box-binding protein (*TBP*) genes were designed and synthesized commercially by Qiagen. *TBP* was used as an internal control gene. The experiments were repeated twice. The results are expressed as fold changes normalized to TBP expression by the ΔΔCt method.

Cell lines and primary cells

Cell lines and primary cells used in Supplementary Fig. 11 are listed in Supplementary Table 3. These cells were cultured following the manufacturer's instructions. For differentiation of THP-1 and U937 cells into macrophages, cells were treated 20 nM phorbol-12-myristate 13-acetate (PMA) for 48 h. Following differentiation, the PMA-containing medium was replaced with fresh medium, and the cells were used immediately.
