# Supplementary material for: Identification of interferon-stimulated genes that attenuate Ebola virus infection
Source: Nat Commun. 2020 Jun 11;11:2953. doi: 10.1038/s41467-020-16768-7 (PMC7289892; doi:10.1038/s41467-020-16768-7)
Supplement: Supplementary file 3 — Reporting Summary [file 41467_2020_16768_MOESM3_ESM.pdf]

nature research

Corresponding author(s): Yoshihiro Kawaoka and Peter J. Halfmann

Last updated by author(s): April 24, 2020

Reporting Summary

Nature Research wishes to improve the reproducibility of the work that we publish. This form provides structure for consistency and transparency in reporting. For further information on Nature Research policies, see Authors & Referees and the Editorial Policy Checklist.

Statistics

For all statistical analyses, confirm that the following items are present in the figure legend, table legend, main text, or Methods section.

n/a	Confirmed	
☐	☒	The exact sample size (*n*) for each experimental group/condition, given as a discrete number and unit of measurement
☐	☒	A statement on whether measurements were taken from distinct samples or whether the same sample was measured repeatedly
☐	☒	The statistical test(s) used AND whether they are one- or two-sided<br>*Only common tests should be described solely by name; describe more complex techniques in the Methods section.*
☐	☒	A description of all covariates tested
☐	☒	A description of any assumptions or corrections, such as tests of normality and adjustment for multiple comparisons
☐	☒	A full description of the statistical parameters including central tendency (e.g. means) or other basic estimates (e.g. regression coefficient) AND variation (e.g. standard deviation) or associated estimates of uncertainty (e.g. confidence intervals)
☐	☒	For null hypothesis testing, the test statistic (e.g. *F*, *t*, *r*) with confidence intervals, effect sizes, degrees of freedom and *P* value noted<br>*Give P values as exact values whenever suitable.*
☒	☐	For Bayesian analysis, information on the choice of priors and Markov chain Monte Carlo settings
☒	☐	For hierarchical and complex designs, identification of the appropriate level for tests and full reporting of outcomes
☒	☐	Estimates of effect sizes (e.g. Cohen's *d*, Pearson's *r*), indicating how they were calculated

Our web collection on statistics for biologists contains articles on many of the points above.

Software and code

Policy information about availability of computer code

Data collection	Infinite M1000 plate reader (TECAN); FluorChem HD2 system (Protein Simple); LSM 510 META confocal microscope (Carl Zeiss); 7900ht Fast Real-time PCR system (Applied Biosystems); QuantStudio 6 Flex (Applied Biosystems); FACS Aria III flow cytometer (BD Biosciences).
Data analysis	The following software was used as indicated in the relevant sections in the methods: ZEN 2009 software (Carl Zeiss); ImageJ 1.48v; QuantStudio real-time PCR Software v1.3 6 Flex (Applied Biosystems); FlowJo software (Tree Star); Microsoft Excel 2016.

For manuscripts utilizing custom algorithms or software that are central to the research but not yet described in published literature, software must be made available to editors/reviewers. We strongly encourage code deposition in a community repository (e.g. GitHub). See the Nature Research guidelines for submitting code & software for further information.

Data

Policy information about availability of data

All manuscripts must include a data availability statement. This statement should provide the following information, where applicable:

- Accession codes, unique identifiers, or web links for publicly available datasets
- A list of figures that have associated raw data
- A description of any restrictions on data availability

The authors declare that data supporting the findings of this study are available within the paper and its supplementary information files.

Field-specific reporting

Please select the one below that is the best fit for your research. If you are not sure, read the appropriate sections before making your selection.

☒ Life sciences ☐ Behavioural & social sciences ☐ Ecological, evolutionary & environmental sciences

Life sciences study design

All studies must disclose on these points even when the disclosure is negative.

Sample size	Sample size is based on our previous experience in this field and on our protocols set up.
Data exclusions	No data was excluded.
Replication	To confirm reproducibility of data presented in the manuscript, all experiments were conducted at least two times unless otherwise indicated in the figure legends.
Randomization	No experimental groups were allocated in this study.
Blinding	Blinding was used for the primary screening in this study.

Reporting for specific materials, systems and methods

We require information from authors about some types of materials, experimental systems and methods used in many studies. Here, indicate whether each material, system or method listed is relevant to your study. If you are not sure if a list item applies to your research, read the appropriate section before selecting a response.

Materials & experimental systems

n/a	Involved in the study	
☐	☒	Antibodies
☐	☒	Eukaryotic cell lines
☒	☐	Palaeontology
☒	☐	Animals and other organisms
☒	☐	Human research participants
☒	☐	Clinical data

Methods

n/a	Involved in the study	
☒	☐	ChIP-seq
☐	☒	Flow cytometry
☒	☐	MRI-based neuroimaging

Antibodies

Antibodies used

Antibodies against EBOV proteins were generated in our laboratory; EBOV VP40 (cl. 6), EBOV GP (cl. 254/3.12), EBOV NP (R5071), EBOV VP35 (cl. 1), EBOV VP30 (cl. 3), EBOV VP24 (cl. 21-5.2.5).
Antibodies used are the following (mAb, clone, supplier name, catalog # and lot #);
Mouse α-beta-actin (N/A, Abcam, ab8224, GR151143-4),
Rabbit α-PFKFB3 (D7H4Q, Cell signaling technology, 13132, 1),
Mouse α-ODC1 (1G1, Origene, TA501546, A01),
Rabbit α-MAP3K5 (D11C9, Cell signaling technology, 8662, 1),
Rabbit α-AKT3 (62A8, Cell signaling technology, 3788, 3),
Mouse α-GBP2 (1E5, Origene, TA500657, A01),
Rabbit α-DCP1A (N/A, Sigma, D5444, 020M4824),
Rabbit α-BTN3A3 (N/A, Sigma, HPA007904, R02562),
Rabbit α-CCDC92 (N/A, Abcam, ab104028, GR95014-1),
Rabbit α-FLT1 (N/A, Origene, TA303515, YJ071908CS),
Mouse α-FLAG tag (M2, Sigma, F3165, 87K60021),
Rabbit α-His tag (D3I1O, Cell signaling technology, 12698, 4)

Validation

Validation of antibodies against EBOV VP40, GP, NP, VP35, VP30, and VP24 is shown in Figures 2a, 3c, 4b, and 5b. We detected these viral proteins at the expected molecular weight from HEK-293T cell lysates after transfection with the appropriate corresponding protein expression vector or from lysates of HeLa cells stably expressing VP30 that were infected with EBOV lacking VP30.

Validation of antibodies against PFKFB3, ODC1, MAP3K5, AKT3, GBP2, DCP1A, BTN3A3, CCDC92, and FLT1 is shown in Supplementary Figure 2. We detected these cellular proteins at the expected molecular weight from HEK-293T cell lysates after transfection with the appropriate corresponding protein expression vector.

Validation of antibodies against FLAG and His tags is shown in Supplementary Figure 8. We detected tagged versions of proteins at the expected molecular weight from HEK-293T cells transfected with the appropriate corresponding protein expression vector.

Validation of antibodies against alpha-tubulin is shown in Figure 2a and Supplementary Figures 1a and 3a, where tubulin from HEK-293T cells, HeLa cells, and Huh7.0 cells was detected at the expected molecular weight. The manufacturer states that the

antibody is specific for alpha-tubulin in immunoblotting assays.

Eukaryotic cell lines

Policy information about cell lines

Cell line source(s)	- Vero cells, Huh7.0 cells, Hela cells, A549 cells, HepG2, U937 cells, THP-1 cells, and Jurkat cells are our laboratory stock.<br>- U-138 MG cells, SH-SY5Y cells, MCF7 cells, NTERA-2 cells, PC-3 cells, U-2 OS cells, and A431 cells were obtained from ATCC.<br>- Astrocytes, HPAEC, InMyoFib, and HUVEC were obtained from Lonza.<br>- Human neural stem cells were obtained from Thermo.<br>- Human mast cells were obtained from Sigma.
Authentication	None of the cell lines were genotypically authenticated in our laboratory
Mycoplasma contamination	All cell lines were tested monthly in our laboratory and were negative each time for mycoplasma contamination.
Commonly misidentified lines (See ICLAC register)	N/A

Flow Cytometry

Plots

Confirm that:

☒ The axis labels state the marker and fluorochrome used (e.g. CD4-FITC).

☒ The axis scales are clearly visible. Include numbers along axes only for bottom left plot of group (a 'group' is an analysis of identical markers).

☒ All plots are contour plots with outliers or pseudocolor plots.

☒ A numerical value for number of cells or percentage (with statistics) is provided.

Methodology

Sample preparation	Transfected and infected HEK-293T cells as described in the supplementary methods section were collected with 0.25% trypsin and fixed with 4% PFA for 10min at room temperature. After washes with PBS, cells were analyzed.
Instrument	FACS Aria III flow cytometer (BD Biosciences)
Software	FlowJo software (Tree Star)
Cell population abundance	No cell sorting was performed.
Gating strategy	FACS analysis was performed on single cell suspensions. In all flow cytometry experiments, cells were gated on FSC/SSC (forward scatter/side scatter) to exclude dead cells and debris.

☒ Tick this box to confirm that a figure exemplifying the gating strategy is provided in the Supplementary Information.
